# Assessment of bone density and microarchitecture in patients with familial partial lipodystrophy

**DOI:** 10.3389/fendo.2026.1691552

**Published:** 2026-05-04

**Authors:** Raquel Beatriz Gonçalves Muniz, Cynthia M. Valerio, Eduardo Medeiros Ferreira da Gama, Bárbara Gehrke Smith, Luiz F. Viola, Monike Cristine Paes Santos Franco Farias, Patrícia Silva Motta Gomes e Silva de Macedo, Rodrigo Oliveira Moreira, Amelio F. Godoy-Matos, Maria Lucia Fleiuss Farias, Miguel Madeira

**Affiliations:** 1 Department of Metabolism, Institute of Diabetes and Endocrinology of Rio de Janeiro (IEDE), Rio de Janeiro, Brazil; 2 Endocrinology Division, Federal University of Rio de Janeiro (UFRJ), Rio de Janeiro, Brazil; 3 Endocrinology Division, Faculty of Medical Sciences, State University of Rio de Janeiro, Rio de Janeiro, Brazil

**Keywords:** Bone microarchitecture, bone mineral density, diabetes, familial partial lipodystrophy, insulin resistance

## Abstract

**Introduction:**

Familial partial lipodystrophy (FPL) is a rare condition related to partial deficiency of adipose tissue and consequent insulin resistance resulting in metabolic alterations. Although type 2 diabetes mellitus is associated with bone changes, few studies have evaluated bone health in familial partial lipodystrophy.

**Methods:**

Cross-sectional study that selected patients from a specific outpatient clinic for LPF and controls from a database of healthy individuals. Patients and controls underwent clinical evaluation and examinations by dual energy X-ray absorptiometry (DXA) and high-resolution quantitative peripheral computed tomography (HR-pQCT).

**Results:**

Twenty-four patients were included, with a mean age of 43.4 years (± 11.9), as twenty controls, with a mean age of 39.4 (± 6.4). In the FPL group, 15 participants had a diagnosis of DM, with a mean disease duration of 16.5 years (± 7.4). Regarding DXA, there was a statistical difference in the Z-score of the spine and femur, with higher values in the FPL group. Evaluating HR-pQCT in the tibia and radius, a significant difference in cortical pore diameter at the radius was observed, with higher values in the FPL group. A comparison between the FPL subgroup with DM and the controls confirmed the difference in porosity diameter and showed a statistical difference in the radius trabecular thickness parameter (Tb.Th), with greater Tb.Th in the FPL group with DM. Two patients presented femur fracture after falling from their own height during the study – after 20 years of diagnosis of DM and additional risk factors for frailty.

**Conclusion:**

In summary, FPL patients - especially those with DM - exhibit alterations in bone microarchitecture, including increased cortical pore diameter and trabecular thickness. Despite normal or even elevated Z-scores on DXA, fragility fractures may occur, warranting routine imaging and preventive strategies in this population.

## 1 Introduction

Lipodystrophies are a heterogeneous group of rare diseases characterized by adipose tissue deficiency. They are divided into partial or generalized forms, according to the degree of adipose tissue deficiency, and familial or acquired forms, according to the etiology (1, 2). FPL is a genetic disorder characterized by a loss of adipose tissue in the lower limbs and gluteal region, often accompanied by fat accumulation in the trunk, face, and neck. Most cases are transmitted in an autosomal dominant manner and manifest during late childhood or shortly after puberty (1, 3, 4).

As a result of the limited expansion capacity of adipose tissue, hypoleptinemia and hypoadiponectinemia are common findings, which, together with fat redistribution and ectopic fat accumulation, contribute to insulin resistance and various metabolic abnormalities (1, 3). However, the genetic background of FPL also predisposes to non-metabolic manifestations, including dilated cardiomyopathy, cardiac conduction abnormalities, and muscular dystrophy (4, 5).

FPL encompasses variants associated with mutations in the *LMNA, PPARG, PLIN1, LIPE, AKT2, MFN2*, and *CAV1* genes. FPL type 1, known as Köbberling syndrome, is considered a polygenic form (3, 6). Among monogenic forms, FPL type 2 is the most common, caused by variants in the *LMNA* gene, accounting for approximately 70% of genetically confirmed cases (7, 8). Patients present with a high prevalence of hypertriglyceridemia and diabetes. Cohorts have found hypertriglyceridemia in 66% to 91% of patients and diabetes in 44% to 77% (6).

Regarding skeletal outcomes, Moreira et al. compared 18 patients with FPL to healthy controls and reported lower bone mineral density (BMD) and Z-scores at the radius in the FPL group, while no difference was observed at the lumbar spine or femur. Trabecular Bone Score (TBS) analysis indicated a higher prevalence of partially degraded or degraded microarchitecture in FPL patients. Although this study shows that insulin resistance is negatively associated with TBS, no data are available regarding fracture risk in FPL (9).

Diabetes mellitus (DM), which is common in FPL, is associated with increased fracture risk, although areal BMD is often preserved. Consequently, Dual Energy X-ray Absorptiometry (DXA) alone may underestimate fracture risk in this population. Advanced imaging techniques, such as high-resolution peripheral quantitative computed tomography (HR-pQCT) and TBS, have shown impaired bone microarchitecture in patients with type 2 diabetes mellitus (T2DM), including increased cortical porosity and trabecular deterioration (10, 11).

This study aims to evaluate bone mineral density and microarchitecture in patients with FPL using DXA and HR-pQCT, due to the scarcity of bone assessments in this patient group. We also aim to explore the relationship between diabetes and metabolic conditions in FPL patients and bone fragility.

## 2 Methods

### 2.1 Study design and population

An observational, cross-sectional study was conducted between May 2022 and December 2024, involving 44 participants: 24 patients diagnosed with FPL and 20 age-, sex-, and BMI-matched controls. All control participants were premenopausal, whereas seven patients in the FPL group were postmenopausal. Patients were recruited from the Institute of Diabetes and Endocrinology of Rio de Janeiro (IEDE), where they are followed in a dedicated lipodystrophy outpatient clinic. Controls were selected from a database of healthy individuals with no metabolic alterations or conditions affecting bone health. Neither the patients nor the control group had a history of smoking. All participants provided written informed consent, and the study protocol was approved by the institutional ethics committee (Protocol Number: 28388720.9.0000.5266).

The diagnosis of FPL was based on clinical identification of partial subcutaneous fat loss, confirmed by thigh skinfold measurement (cut-off <22 mm in females), in association with metabolic abnormalities. Genetic testing was subsequently performed using a panel for hypertriglyceridemia and pancreatitis-related genes (12). The panel included the following genes: *ABCA1, AGPAT2, AKT2, APOA5, APOC2, BSCL2, CAV1, CAVIN1, CFTR, CIDEC, CTRC, CYP27A1, GPIHBP1, LIPA, LIPE, LMF1, LMNA, LMNB2, LPL, MFN2, PLIN1, POLD1, PPARG, PRSS1, PSMB8, SMPD1, SPINK1*, and *ZMPSTE24.*

Genetic variants were identified as follows: *LMNA* (n = 10), *LPL* heterozygous (n = 4), *PPARG* (n = 3), and combined *LMNA* + *AKT2* (n = 2). Rare variants were identified in *MFN2, BSCL2* (heterozygous), and *SPINK1* (each in 1 patient). Two patients had no pathogenic mutations identified.

Collected clinical data included age, weight, height, BMI, presence of metabolic disorders, and use of medications known to affect bone metabolism, including pioglitazone.

Exclusion criteria were: age <18 years, known bone metabolic disorders, or confounding conditions such as hyperparathyroidism, hyperthyroidism, or current glucocorticoid use. Male participants were excluded, due to the disproportionate number of men and women, with few men being monitored in our service.

### 2.2 Laboratory evaluation

All participants underwent routine laboratory testing, including: fasting glucose, glycated hemoglobin (HbA1c), total cholesterol and fractions, calcium, phosphorus, 25-hydroxyvitamin D [25(OH)D], parathyroid hormone (PTH), thyroid-stimulating hormone (TSH), and free thyroxine (FT4).

HbA1c was measured by high-performance liquid chromatography (HPLC). Glucose and cholesterol levels were measured using enzymatic assays. PTH, 25(OH)D, TSH, and FT4 levels were determined by chemiluminescence, while calcium and phosphorus were measured using the colorimetric method. Reference range for PTH: 12–88 pg/mL.

### 2.3 Dual energy x-ray absorptiometry

Bone density and body composition assessments were performed using a GE Lunar Prodigy Advance (GE Healthcare, Madison, WI, USA). All exams were performed on the same device and analyzed by the same physician.

Bone sites evaluated: radius (33%), lumbar spine (L1–L4), total hip, and femoral neck. Areal BMD was not assessed in postmenopausal women per ISCD recommendations. Z-scores were calculated using age-matched reference data. A Z-score ≤ -2.0 standard-deviation (SD) was considered “below expected for age” as per ISCD. Coefficient of variation (CV) at the institution: 2.3% for the femur and 1.8% for the lumbar spine.

Body composition parameters included: total fat and lean mass (g), fat percentage, appendicular lean mass (Baumgartner Index), fat mass index (FMI), fat mass ratio (FMR; trunk fat %/leg fat %), and proportion of leg fat relative to total fat.

In the FPL group, vertebral fracture assessment (VFA) was performed to screen for morphometric vertebral fractures using Genant’s semiquantitative method. Fractures were graded as mild (Grade 1, 20–25% height loss), moderate (Grade 2, 25–40%), or severe (Grade 3, >40%) (13).

### 2.4 High-resolution quantitative peripheral computed tomography

Volumetric density and bone microarchitecture analysis were performed using HR-pQCT scans, obtained at the distal radius and distal tibia according to standardized acquisition protocols (14). The XtremeCT device (SCANCO Medical AG, Brüttisellen, Switzerland) was used, which uses a 2-dimensional detector combined with a 0.08 mm focal X-ray tube, allowing acquisition of numerous sessions with 82 µm resolution. At each site, 110 slices were obtained, forming a 3-dimensional representation in the axial direction. The metrics analyzed included: total volumetric (Tt.BMD), cortical (Ct.BMD) and trabecular (Tb.BMD) BMD, cortical thickness (Ct.Th), trabecular number (Tb.N), trabecular bone volume fraction (BV/TV), trabecular number (Tb.N), trabecular thickness (Tb.Th), trabecular spacing (Tb.Sp), standard deviation of 1.Tb.N, measuring the inhomogeneity of the trabecular space (Tb.1/N.SD), cortical porosity (Ct.Po), cortical pore diameter (Ct.Po.Dm). The coefficient of variation of the institution is assumed to be 3 to 5% for microarchitectural parameters and 1 to 2% for density measurements.

### 2.5 Statistical analysis

Analyses were conducted using Jamovi software (v2.3.28). Parametric data were analyzed with Student’s t-test; nonparametric data with Mann–Whitney U test. Descriptive statistics were reported as mean ± SD (parametric) or median (min–max) (nonparametric). A p-value <0.05 was considered significant. Pearson or Spearman correlation tests were used depending on variable distribution.

## 3 Results

Twenty-four patients were evaluated in the FPL group, and no statistically significant differences in age or body mass index (BMI) were observed between groups. Serum calcium, phosphorus, and 25(OH)D levels were also comparable. However, PTH levels were significantly higher in the control group (p = 0.002), although values remained within the reference range in both groups. Notably, no FPL patients had elevated PTH, while only one control subject showed a high PTH value (93.1 pg/mL), associated with low vitamin D (25(OH)D = 20 ng/mL) and normal calcium (9 mg/dL) (**Table 1**).

**Table 1:** Anthropometric and laboratory characteristics of the FPL and control groups.

Anthropometric and laboratory characteristics	Reference values	FPL group	Control group	*P-value*
Age (years)	–	43.4 (± 12)	39.4 (± 6.4)	0.185
Weight (kg)	–	70 (54 – 116)	70.9 (54 – 101)	0.832
Height (cm)	–	164 (± 0.05)	162 (± 0.06)	0.276
BMI (kg/m²)	–	25.3 (22.5 – 39.2)	25.6 (22 – 37.6)	0.506
Serum calcium (mg/dl)	8.5 – 10.5	9.5 (± 0.4)	9.6 (± 0.4)	0.234
Serum phosphorus (mg/dl)	2.5 – 5.5	3.8 (± 0.5)	3.9 (± 0.5)	0.794
25 (OH) vit D (ng/ml)	> 20	28.4 (12.6 – 52.8)	21.8 (13.5 – 48)	0.053
PTH (pg/ml)	12 – 88	24 (9 – 66)	44.7 (16.1 – 93.1)	**0.002**
HbA1c (%)	< 5.7	6.7 (5.1 – 12.8)	5.2 (4.5 – 6)	**0.003**
Triglycerides (mg/dl)	< 150	174 (85 – 1012)	70 (21 – 157)	**< 0.001**

Values are expressed as median (minimum and maximum) or mean (standard deviation). 25(OH)vit D, 25-hydroxy-vitamin D; BMI, body mass index; FPL, familial partial lipodystrophy; PTH, parathyroid hormone. The p-values ​​in bold highlight the results with statistical significance.

**Figure 1:**
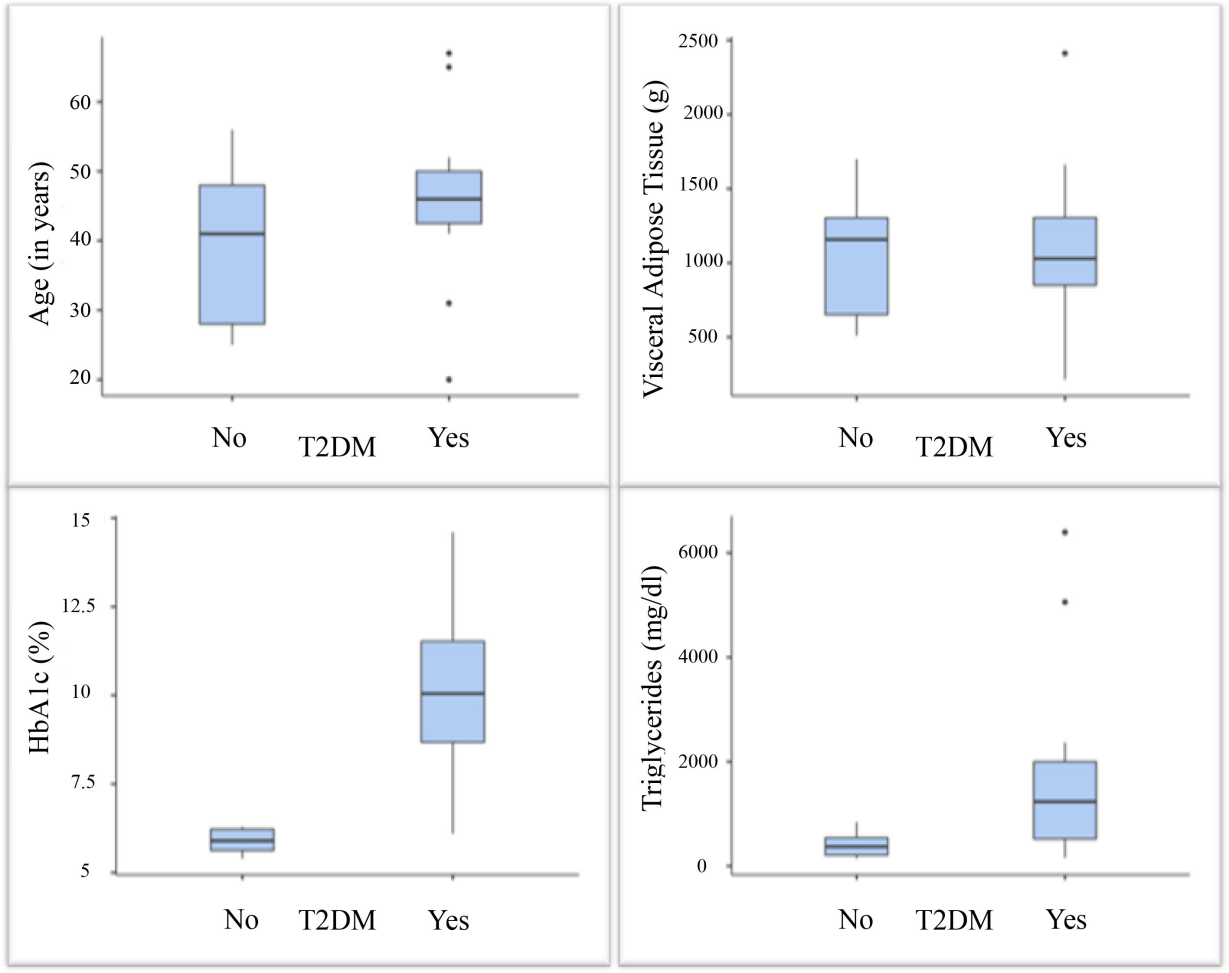
This illustration shows the differences in FPL patients with and without DM regarding age, visceral adipose tissue, and the highest recorded HbA1c and triglyceride levels.

Fifteen FPL participants were diagnosed with DM, with a mean disease duration of 16.5 years (± 7.4). Of these fifteen patients with DM, four are postmenopausal, 80% were on treatment with pioglitazone and 66.6% were on insulin. To characterize the metabolic profile, the highest recorded HbA1c and triglyceride levels were used for comparison between FPL patients with and without DM. Patients without DM showed significantly lower triglyceride levels (p = 0.028) than those with DM. Median triglyceride level in the DM group was 1234 mg/dL (range: 155–6397), compared to 371 mg/dL (range: 143–840) in the group without DM. Mean HbA1c in patients with DM was 10.2% (± 2.2), versus 5.89% (± 0.3) in the group without DM. In **Figure 1**, we present an illustration highlighting differences in HbA1c, triglycerides and age between these two subgroups of FPL patients. To compare the FPL and control groups, we included the most recently recorded HbA1c and triglyceride levels in **Table 1**.

Regarding DXA results, no significant difference was observed in the radius Z-score between groups. However, spine and total femur Z-scores were significantly higher in the FPL group (p = 0.040 and p = 0.021, respectively). Subgroup analysis confirmed that FPL patients with DM had significantly higher spine (p = 0.006) and femur Z-scores (p = 0.011) compared to controls (**Table 2**).

**Table 2:** Bone and whole-body densitometry assessment in the FPL and control groups.

DXA	FPL group	Control group	*P-value*
Lumbar spine Zs	+ 0.7 (± 1.0)	+ 0.1 (± 0.9)	**0.040**
Total femur Zs	+ 0.4 (± 1.1)	- 0.3 (± 0.7)	**0.021**
Radius 33% Zs	- 0.6 (± 1.1)	- 0.5 (± 0.8)	0.728
Muscle mass (g)	48,262 (± 7,642)	40,934 (± 4351)	**< 0.001**
Fat mass (g)	19,049 (10,925 – 41,404)	28,867 (18,630 – 46,429)	**0.002**
FMR	1.5 (± 0,2)	0.9 (± 0,1)	**<0.001**
Lower limbs fat mass/total fat mass (%)	21 (15 – 30)	37 (31 – 45)	**<0.001**
VAT mass (g)	1093 (± 92)	508 (± 88)	**<0.001**
Fat percentage (%)	29.6 (± 1.6)	40.4 (± 1.1)	**<0.001**
FMI (kg/m²)	7.85 (3.91 – 15.5)	10.8 (6.59 – 17.3)	**0.001**
BI (kg/m^2^)	7.57 (1.0 – 10.8)	7.31 (5.36 – 9.03)	0.370

Values are expressed as median (minimum and maximum) or mean (standard deviation). FPL, familial partial lipodystrophy; BMD, bone mineral density; Zs, Z-score; g, grams; VAT, visceral adipose tissue; FMI, Fat mass index; BI, Baumgartner index. The p-values ​​in bold highlight the results with statistical significance.

In body composition analysis, the FPL group had significantly greater lean mass and visceral fat, but lower total fat mass and fat percentage. The Fat Mass Index (FMI) and lower-limb fat percentage (relative to total fat) were also significantly lower in the FPL group. No significant difference was found in the Baumgartner index between groups. FMR (fat mass ratio) was significantly higher in FPL patients, indicating greater central adiposity (**Table 2**).

Bone microarchitecture was assessed using HR-pQCT at the distal tibia and radius. A significant difference in cortical pore diameter at the radius was observed, with higher values in the FPL group (mean 0.155 mm vs. 0.144 mm; p = 0.027). No differences were found in other microarchitectural parameters between groups (**Table 3**).

**Table 3:** Assessment of bone microarchitecture of distal radius and tibia in patients with FPL and control group.

HR-pQCT	FPL group	Control group	*P-value*
Distal radius (volumetric parameters)			
Tt.Ar (mm²)	263 (± 51)	243 (± 41.7)	0.191
Ct.Ar (mm²)	59 (± 12.5)	53.8 (± 11.9)	0.190
Tb.Ar (mm²)	200 (± 46.3)	186 (± 43)	0.323
Distal radius (cortical parameters)			
Ct.Th (mm)	0.857 (± 0.171)	0.821 (± 0.204)	0.552
Ct.Pm (mm)	69 (± 7.46)	66.2 (± 6.55)	0.214
Ct.Po (%)	1.0 (0.5 – 6.9)	0.8 (0.4 – 1.9)	0.283
Ct.Po.Dm (mm)	0.155 (± 0.01)	0.144 (± 0.01)	**0.027**
Distal radius (trabecular parameters)			
BV/TV (%)	0.143 (± 0.03)	0.132 (± 0.02)	0.344
Tb.N (1/mm)	1.95 (0.81 – 2.50)	1.90 (1.50 – 2.34)	0.844
Tb.Th (mm)	0.07 (0.05 – 0.12)	0.06 (0.04 – 0.11)	0.221
Tb.Sp (mm)	0.43 (0.30 – 1.1)	0.46 (0.35 – 0.58)	0.747
Tb.1/N.SD (mm)	0.18 (0.11 – 0.96)	0.19 (0.13 – 0.34)	0.546
Distal tibia (volumetric parameters)			
Tt.Ar (mm²)	668 (± 135)	625 (± 100)	0.276
Ct.Ar (mm²)	115 (± 27)	121 (± 16.1)	0.389
Tb.Ar (mm²)	548 (± 131)	502 (± 102)	0.231
Distal tibia (cortical parameters)			
Ct.Th (mm)	1.14 (± 0.27)	1.25 (± 0.2)	0.182
Ct.Pm (mm)	101 (± 9.6)	97 (± 7.9)	0.223
Ct.Po (%)	3.8 (1.2 – 11.1)	2.7 (1.1 – 7.4)	0.185
Ct.Po.Dm (mm)	0.180 (± 0.03)	0.180 (± 0.02)	1.000
Distal tibia (trabecular parameters)			
BV/TV (%)	0.130 (± 0.02)	0.129 (± 0.02)	0.919
Tb.N (1/mm)	1.81 (± 0.3)	1.77 (± 0.3)	0.748
Tb.Th (mm)	0.07 (± 0.1)	0.07 (± 0.1)	0.752
Tb.Sp (mm)	0.472 (0.340 – 0.861)	0.472 (0.381 – 0.930)	0.759
Tb.1/N.SD (mm)	0.217 (0.133 – 0.451)	0.212 (0.158 – 0.513)	1.000

Values are expressed as median (minimum and maximum) or mean (standard deviation).FPL, familial partial lipodystrophy; Tt.Ar, total area; Ct.Ar, cortical area; Tb.Ar, trabecular area; Ct.Th, cortical thickness; Ct.Pm, cortical perimeter; Ct.Po, cortical porosity; Ct.Po.Dm, cortical pore diameter; tBV/TV, bone volume-to-total volume ratio; Tb.N, trabecular number; Tb.Th, trabecular thickness; Tb.Sp, trabecular separation; Tb.1/N.SD, inhomogeneity of trabecular network. The p-values ​​in bold highlight the results with statistical significance.

Subgroup analysis comparing FPL patients with DM and controls confirmed the difference in cortical pore diameter (0.156 mm vs. 0.144 mm; p = 0.029) and revealed significantly greater trabecular thickness (Tb.Th) at the radius in the FPL + DM group (median 0.078 mm vs. 0.065 mm; p = 0.048). Correlations between clinical and densitometric data in the FPL group revealed no significant associations between spine or femur Z-scores and BMI, waist circumference, triglyceride levels, or HbA1c. Similarly, no correlations were observed with FMI, lean or fat mass, fat percentage, or visceral adipose.

Correlations between clinical and densitometric data in the FPL group revealed no significant associations between spine or femur Z-scores and BMI, waist circumference, triglyceride levels, or HbA1c. Similarly, no correlations were observed with FMI, lean or fat mass, fat percentage, or visceral adipose tissue. However, a significant positive correlation was found between radius trabecular thickness (Tb.Th) and the highest HbA1c value (p = 0.004; Spearman’s R = 0.479).

At baseline, two patients presented with asymptomatic vertebral fractures identified by VFA: one aged 29 (Grade 1 fracture at L4), and one aged 67 (Grade 2 at L1). Both later experienced fragility fractures of the femur following falls from standing height. The younger patient had normal Z-scores, while the older patient had a Z-score of –2.7 SD at the radius. Both presented normal levels of hemoglobin.

## 4 Discussion

Our study demonstrated increased cortical pore diameter (Ct.Po.Dm) at the radius in patients with FPL, as measured by HR-pQCT. This finding persisted in the subgroup with DM. In the subgroup with DM, we also observed greater trabecular thickness (Tb.Th) at the radius compared to controls. DXA results revealed significantly higher spine and femur Z-scores in FPL patients compared to healthy controls.

Although the control group had higher PTH levels than the FPL group, the median values for both groups were within the reference range. Only one patient in the control group had elevated PTH levels. Despite hyperparathyroidism being known to increase bone resorption and alter cortical and trabecular microarchitecture, the main evidence points to the impact of elevated PTH levels (15). Considering the superior cortical microarchitecture of the control group compared to the FPL group, and the absence of difference in radius density between the two groups, the observed changes appear independent of PTH levels.

One of the hallmarks of FPL is severe insulin resistance due to impaired expandability of subcutaneous adipose tissue, resulting in ectopic fat accumulation and decreased leptin and adiponectin levels (8). A Brazilian multicenter study of 106 genetically confirmed lipodystrophy patients (mean age 44 ± 15 years, 78.3% female) reported high prevalence and severity of metabolic abnormalities, including DM in 57.5% of patients and hypertriglyceridemia (>500 mg/dL) in 34.9% (16). In our FPL cohort, DM was identified in 62.5% of patients, of whom 66.6% had HbA1c >8%. Hypertriglyceridemia >150 mg/dL was seen in 91.6%, and >500 mg/dL in 62.5%, reflecting a severe metabolic phenotype.

Body composition analysis showed higher lean mass but lower fat mass and FMI in the FPL group. Visceral fat was increased, supporting the presence of insulin resistance (8). Notably, the Baumgartner index did not differ significantly between groups. In view of the high prevalence of diabetes and insulin resistance in FPL patients, it is important to note that this combination of greater trabecular bone and deterioration in cortical microarchitecture has also been observed in other HR-pQCT studies of patients with T2DM (10).

The impact of insulin resistance on bone has been evaluated in postmenopausal women without T2DM. In one HR-pQCT study, higher HOMA-IR was associated with increased trabecular and cortical BMD and thickness at both radius and tibia after weight adjustment (17). Although insulin resistance may initially enhance bone mass, our findings suggest a more complex interaction. The increase in trabecular thickness observed only in FPL patients with DM may reflect a compensatory response to chronic hyperinsulinemia. Interestingly, this subgroup also had the highest triglyceride levels (median 1234 mg/dL), suggesting a link between severe metabolic disturbance and altered bone architecture (18). Shanbhogue et al. postulated that insulin resistance may initially favor bone quality, but that prolonged exposure, glycemic deterioration, and microvascular complications eventually lead to bone fragility (17).

Multiple factors contribute to bone fragility in T2DM, including insulin therapy, poor glycemic control, long disease duration, and the use of thiazolidinediones (e.g., pioglitazone) (10). In our study, 80% of FPL patients with DM were on pioglitazone, and 66.6% on insulin. Among FPL patients without DM, only one used pioglitazone to manage steatohepatitis.

A database study of 206,672 people with T2DM assessed the cumulative effect of pioglitazone on the risk of hip fractures. While initial studies indicated that pioglitazone impacted only the risk of distal fractures in women, this study revealed an increased risk of hip fractures in both men and women associated with the cumulative effect of pioglitazone use (19). A higher risk of fractures is also linked to insulin therapy, probably because of the greater chance of hypoglycemia (20).

HR-pQCT studies in populations with T2DM have demonstrated increased cortical porosity with preserved cortical thickness. Similarly, TBS analysis often reveals microarchitectural deterioration, especially in those with poor glycemic control (9, 10). A recent study evaluating HR-pQCT in T2DM found that cortical pore diameter (Ct.Po.Dm) was increased in patients with DM, while total cortical porosity (Ct.Po) was only elevated in those with microvascular complications. This supports the notion that Ct.Po.Dm may represent an early marker of bone remodeling defects linked to hyperglycemia (21). Our findings reinforce this hypothesis, given that Ct.Po.Dm differed between groups.

Compared to Moreira et al., who found lower radius Z-scores in FPL patients, our study differs in that we included a broader spectrum of FPL variants, not limited to LMNA mutations, and had a slightly lower prevalence of DM (9). These differences may partially explain the discordance in BMD results.

Despite only subtle HR-pQCT changes, two patients suffered fragility femoral fractures during the study. Both had vertebral fractures detected on baseline VFA and carried rare FPL variants (BSCL2 and MFN2). The 29-year-old had early-onset DM and irritable bowel syndrome; the 67-year-old had reduced mobility due to lower limb weakness. Both were in use of pioglitazone. These cases highlight that fractures may occur even in the presence of preserved BMD and underscore the importance of clinical vigilance and VFA screening in FPL. The VFA findings and observed fragility fractures support recommendations to perform spinal imaging in DM patients, especially in high-risk groups like FPL. Vertebral fractures independently increase hip fracture risk and are often clinically silent (10, 11).

This study is limited by the small sample size and phenotypic heterogeneity, which reflects the rarity and genetic diversity of FPL. The inclusion of multiple genetic variants precludes conclusions regarding genotype-specific bone effects. It was not possible to determine whether the bone changes were caused by FPL, diabetes or pioglitazone use. Possible factors that could interfere with bone microarchitecture, such as blood metal levels, were not evaluated either (22). Also, it would be interesting to have the analysis of bone marrow adipose tissue, considering the scarcity in generalized forms of lipodystrophy and only a few evidence suggesting preservation or higher bone marrow adipose tissue in FPL (9).

In summary, FPL patients exhibit alterations in bone microarchitecture, particularly an increase in cortical pore diameter. These findings likely reflect the combined effects of insulin resistance, chronic hyperglycemia, and medication use. Despite subtle changes in bone microarchitecture and normal or even elevated Z-scores on DXA, fragility fractures may occur, warranting routine imaging and preventive strategies in this population. Further longitudinal studies are needed to confirm these findings and elucidate the mechanisms linking lipodystrophy, bone quality, and fracture risk.

## Data Availability

The datasets for this article are not publicly available due to concerns regarding participant anonymity. Requests to access the datasets should be directed to the corresponding author.
